# ERK1/2 inhibitors act as monovalent degraders inducing ubiquitylation and proteasome-dependent turnover of ERK2, but not ERK1

**DOI:** 10.1042/BCJ20220598

**Published:** 2023-05-04

**Authors:** Kathryn Balmanno, Andrew M. Kidger, Dominic P. Byrne, Matthew J. Sale, Nejma Nassman, Patrick A. Eyers, Simon J. Cook

**Affiliations:** 1 Signalling Programme, The Babraham Institute, Babraham Research Campus, Cambridge CB22 3AT, U.K.; 2 Department of Biochemistry and Systems Biology, Institute of Systems, Molecular and Integrative Biology, University of Liverpool, Liverpool, U.K.

**Keywords:** ERK inhibitors, extracellular signal-regulated kinases, MEK, RAF, RAS, ubiquitin proteasome system

## Abstract

Innate or acquired resistance to small molecule BRAF or MEK1/2 inhibitors (BRAFi or MEKi) typically arises through mechanisms that sustain or reinstate ERK1/2 activation. This has led to the development of a range of ERK1/2 inhibitors (ERKi) that either inhibit kinase catalytic activity (catERKi) or additionally prevent the activating pT-E-pY dual phosphorylation of ERK1/2 by MEK1/2 (dual-mechanism or dmERKi). Here, we show that eight different ERKi (both catERKi or dmERKi) drive the turnover of ERK2, the most abundant ERK isoform, with little or no effect on ERK1. Thermal stability assays show that ERKi do not destabilise ERK2 (or ERK1) *in vitro*, suggesting that ERK2 turnover is a cellular consequence of ERKi binding. ERK2 turnover is not observed upon treatment with MEKi alone, suggesting it is ERKi binding to ERK2 that drives ERK2 turnover. However, MEKi pre-treatment, which blocks ERK2 pT-E-pY phosphorylation and dissociation from MEK1/2, prevents ERK2 turnover. ERKi treatment of cells drives the poly-ubiquitylation and proteasome-dependent turnover of ERK2 and pharmacological or genetic inhibition of Cullin-RING E3 ligases prevents this. Our results suggest that ERKi, including current clinical candidates, act as ‘kinase degraders’, driving the proteasome-dependent turnover of their major target, ERK2. This may be relevant to the suggestion of kinase-independent effects of ERK1/2 and the therapeutic use of ERKi.

## Introduction

The RAS-regulated RAF-MEK1/2-ERK1/2 signalling pathway drives cell survival, cell proliferation and differentiation in response to extracellular stimuli [1]. Receptor-driven activation of the RAS GTPases results in the dimerisation and activation of RAF kinases (ARAF, BRAF and CRAF) [2,3] which phosphorylate conserved serine residues in the activation loop of MEK1/2, resulting in their activation. Active MEK1/2 in turn activate ERK1/2 by phosphorylating threonine and tyrosine residues in the T-E-Y motif in the ERK1/2 activation loop (pT-E-pY or p-ERK1/2) [4]. This promotes the release of activated ERK1/2 from MEK1/2, enabling ERK1/2 to phosphorylate cytoplasmic substrates, such as the kinase RSK, or enter the nucleus to phosphorylate transcription factors to regulate gene expression [1,5]. Depending on the magnitude and duration of activation, ERK1/2 can also promote cell proliferation, cell cycle arrest, senescence and even cell death [6]. ERK1/2 activity is also controlled by negative feedback loops [7] including direct inhibitory phosphorylation of upstream pathway components such as the RAF proteins by ERK1/2 [8], and the *de novo* expression of MAP kinase phosphatases [9].

In a range of human cancers activating mutations in receptor tyrosine kinases (RTKs), RAS, BRAF and more rarely CRAF and MEK1/2 results in de-regulated ERK1/2 activation [10] that drives the acquisition and maintenance of key cancer hallmarks. Small-molecule BRAF and MEK1/2 inhibitors (RAFi and MEKi) are now approved and used in the clinic to treat BRAF-mutant melanoma [11,12] and BRAF-mutant colorectal cancer [13]. In addition, the MEKi selumetinib has been approved for paediatric neurofibromatosis [14], a disease driven by RAS activation resulting from mutations in NF1. Innate or acquired resistance to RAFi and/or MEKi is common: innate resistance can occur through relief of negative feedback resulting in the restoration of ERK1/2 activity, whilst acquired resistance to RAFi or MEKi emerges through a range of mechanisms (BRAF amplification, MEK mutation, etc.) that also reinstate ERK1/2 signalling [11,12,15]. This validates the use of ERKi to forestall or overcome acquired resistance and various ERKi are now undergoing clinical evaluation [16,17]. These ERKi include those that inhibit catalytic activity (catERKi, such as BVD-523) and those that additionally prevent the activating phosphorylation of ERK1/2 by MEK1/2 (dual-mechanism or dmERKi, such as SCH772984).

In the course of investigating the biological activity of different ERKi we noted that they caused a progressive decrease in the abundance of ERK1/2 [17]. Here we show that eight different ERKi (both catERKi or dmERKi) drive the poly-ubiquitylation and proteasome-dependent degradation of ERK2, the most abundant ERK isoform. ERKi-induced loss of ERK2 is dependent upon Cullin-RING E3 ligases. These results may be relevant to the proposed kinase-independent effects of ERK1/2 [18–22] and the therapeutic use of ERKi.

## Results

### ERK1/2 inhibitors decrease the abundance of ERK2 but not ERK1

**Figure 1.**
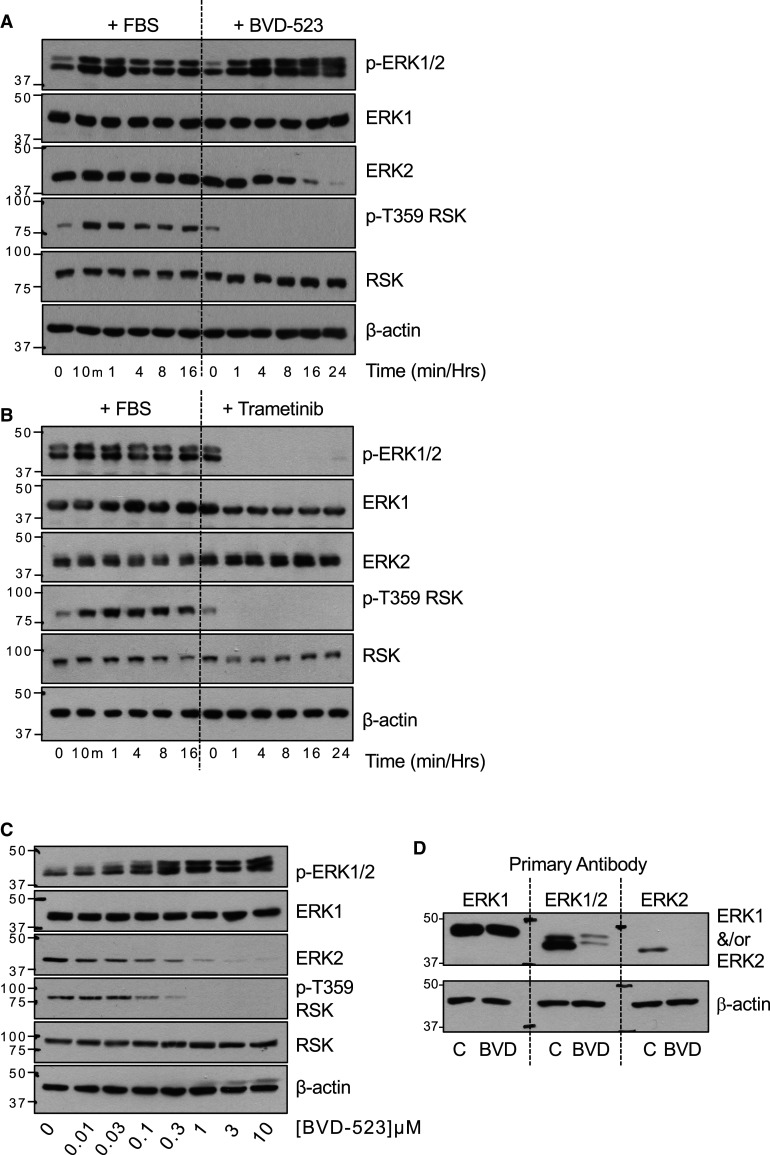
The ERK1/2 inhibitor BVD-523 decreases the abundance of ERK2, but not ERK1. (**A**) HCT116 cells were serum starved for 24 h and then restimulated by addition of 10% (v/v) FBS or were left in complete medium and treated with 3 µM BVD-523 for the indicated times. (**B**) HCT116 cells were serum starved for 24 h and then restimulated by addition of 10% (v/v) FBS or were left in complete medium and treated with 100 nM Trametinib for the indicated times. (**C**) HCT116 cells in complete medium were treated with the indicated concentrations of BVD-523 for 24 h. (**D**) HCT116 cells maintained in complete medium were treated with 3 µM BVD-523 for 24 h. In (**A**–**C**) whole cell lysates were fractionated by SDS–PAGE before immunoblotting with the indicated antibodies. In (**D**) the same control and BVD-523-treated lysates were resolved on a single SDS–PAGE gel that was transferred to PVDF, cut and probed with the indicated ERK1, ERK1/2 or ERK2 antibodies. Results are shown from a single experiment; three experiments gave identical results. Molecular masses in kDa are indicated on the left-hand side.

In the course of investigating the mechanism of action of different ERKi we noted that they caused a progressive decrease in the abundance of ERK1/2 [17]. To investigate this in further detail, serum starved HCT116 cells were re-stimulated with FBS, which led to the characteristic activation of ERK1/2 as monitored by increases in dual phosphorylation of ERK1/2 at pT-E-pY (p-ERK1/2) and p-T359-RSK, an ERK1/2 substrate; the total abundance of ERK1 or ERK2 was unchanged (Figure 1A). In parallel, treatment of HCT116 cells with the catERKi BVD-523 caused the immediate loss of p-T359-RSK but a gradual increase in p-ERK1/2 levels. This is because BVD-523 inhibits ERK1/2 catalytic activity but not its pT-E-pY phosphorylation by MEK1/2 [17]; consequently, in cells wild type BRAF (e.g. HCT116 with KRAS^G13D^) loss of ERK1/2-mediated inhibitory feedback phosphorylation of RAF drives reactivation of MEK1/2 and thence ERK1/2. Strikingly, we also observed that BVD-523 caused a gradual decline in ERK2 abundance that was maximal after 16–24 h of BVD-523 treatment, whereas ERK1 and RSK abundance were unaffected (Figure 1A). We repeated these experiments using Trametinib, a MEKi, instead of BVD-523. Trametinib caused the immediate loss of both p-ERK1/2 and p-T359-RSK indicating that MEK1/2 were inhibited upstream of ERK1/2. In contrast with BVD-523, Trametinib had no effect on the abundance of ERK2 (or ERK1) (Figure 1B). These results indicated that classical methods to increase (FBS) or decrease (MEKi Trametinib) levels of p-ERK1/2 had no effect on total ERK1/2 abundance, in line with multiple previous studies; in contrast, BVD-523 exposure caused a selective loss of ERK2 protein. The BVD-523-driven loss of ERK2 was dose-dependent, tracking closely with the loss p-T359-RSK. (Figure 1C). Selective loss of ERK2 but not ERK1 following BVD-523 treatment was confirmed by western blotting the same cell extracts with an ERK1-specific, a pan-ERK1/2 antibody or an ERK2-specific antibody (Figure 1D).

**Figure 2.**
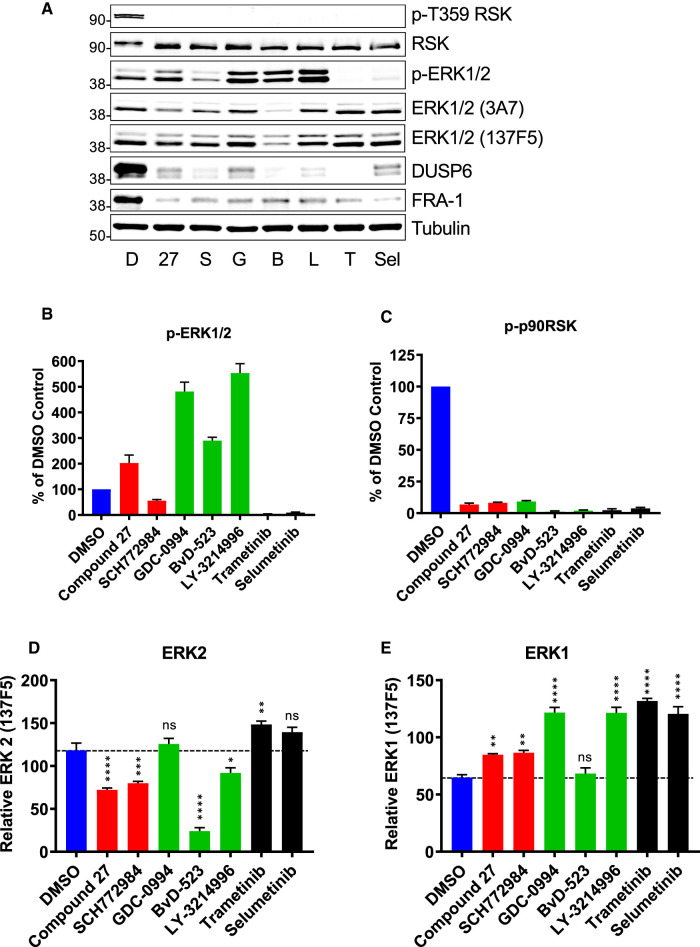
Multiple ERK1/2 inhibitors promote the selective loss of ERK2. (**A**) HCT116 cells maintained in complete medium were treated with DMSO (D) or the ERK inhibitors Compound 27 (27, 100 nM), SCH772984 (S, 100 nM), GDC-0994 (G, 3 µM), BVD-523 (B, 3 µM), LY-3214996 (L, 2 µM) or the MEK inhibitors Trametinib (T, 10 nM) or Selumetinib (S, 100 nM) for 24 h before whole cell lysates were fractionated by SDS–PAGE, immunoblotted with the indicated antibodies and images captured for Li-Cor quantification. Results are shown from a single experiments representative of four others. Molecular masses in kDa are indicated on the left-hand side. (**B**–**E**) Quantitative western blot analysis for (**B**) p-ERK1/2 (pThr202/Tyr204-ERK1/2), (**C**) p-Thr539 p90RSK, (**D**) ERK2 (with ERK1/2 clone 137F5) and (**E**) ERK1 (with ERK1/2 clone 137F5). Results are the mean normalised blot quantification ± SEM, *n* = 4 experiments *P*-values *P* < 0.05 **, *P* < 0.01 *** *P* < 0.001 **** using one-way analysis of variance with Tukey's multiple comparison test comparing each inhibitor to DMSO control.

To determine if ERK2 loss was a general property of ERKi we compared BVD-523, GDC-0994 and LY-3214996 (all catERKi) with SCH772984 and Compound 27 (both dmERKi) [17]; Compound 27 is a precursor of the clinical candidate ASTX029 [23]. For this comparison, we employed quantitative western blotting with two different ERK1/2 antibodies (3A7 and 137F5) combined with Li-Cor detection (Figure 2A,B–E). All ERKi were effective at inhibiting ERK1/2 catalytic output (loss of p-T359 RSK) in HCT116 cells (Figure 2C). Consistently, all three cat ERKi (BVD-523, GDC-0994 and LY-3214996) also increased p-ERK1/2 (Figure 2B), indicating relief of feedback inhibition and re-activation of MEK1/2. This effect was less apparent with the dmERKi Compound 27 and SCH772984, as previously described (Figure 2B) [17]. LiCor detection allowed us to selectively quantify abundance of ERK1 (top band) and ERK2 (bottom band) using the monoclonal pan-ERK1/2 antibody 137F5. Four of five ERKi caused a consistent reduction in ERK2 abundance in HCT116 cells with the rank order BVD-523 > Compound 27 = SCH772984 > LY-3214996 (Figure 2D) whereas there was no reduction in ERK1 abundance (Figure 2E). BVD-523 caused a pronounced 80–90% reduction in ERK2 (Figure 2D), and this was apparent with both pan-ERK1/2 antibodies (Figure 2A). Moreover, neither MEKi (Selumetinib or Trametinib) caused a reduction in ERK2 abundance although they were clearly effective at preventing ERK1/2 activation, judged by the complete loss of p-ERK1/2 and p-T359-RSK (Figure 2B,C).

Using BVD-523 and SCH772984 as comparators we also observed selective loss of ERK2 in response to three other chemically distinct catERKi, VTX-11e, AZ7370 and AZ6197 in BRAF^V600E^ mutant COLO205 cells (Supplementary Figure S1A,B). The only ERKi that failed to drive loss of ERK2 was FR180204; however, this compound caused little loss of pT359 RSK or SPRY2 expression and only a very weak up-regulation of BIM suggesting that its failure to drive loss of ERK2 was due to low affinity towards ERK2. The effect of BVD-523 and SCH772984 on ERK2 abundance was also dose-dependent in COLO205 cells and tracked closely with loss of p-T359-RSK and up-regulation of BIM, markers of ERK engagement and/or inhibition (Supplementary Figure S2).

We expanded our LiCor quantification analysis to include melanoma (A375) and colorectal (HT29) cells with BRAF^V600E^ (Supplementary Figure S3) and a pancreatic cancer cell line with KRAS mutation (Capan-1) (Supplementary Figure S4). In all three cell lines BVD-523, Compound 27, SCH772984 and GDC-0994 caused a significant decrease in ERK2 abundance, whilst LY-3214996 also caused a significant reduction in ERK2 in A375 and Capan-1 cells. Again, the MEKi Trametinib failed to cause a reduction in ERK1/2 abundance.

In summary, eight of nine ERKi tested caused a reduction in the abundance of ERK2, but not ERK1. Loss of ERK2 was not observed in response to treatment with MEKi that prevent ERK1/2 pT-E-pY dual phosphorylation but do not bind directly to ERK1/2, suggesting that ERKi binding was critical for loss of ERK2. Furthermore, loss of ERK2 was observed at the same doses of ERKi that inhibit ERK activity (loss of p-T359-RSK and SPRY2 and/or increases in BIM_EL_ abundance), suggesting that it reflected ERKi binding to ERK2.

### ERKi bind, but do not destabilise ERK2 *in vitro*

**Figure 3.**
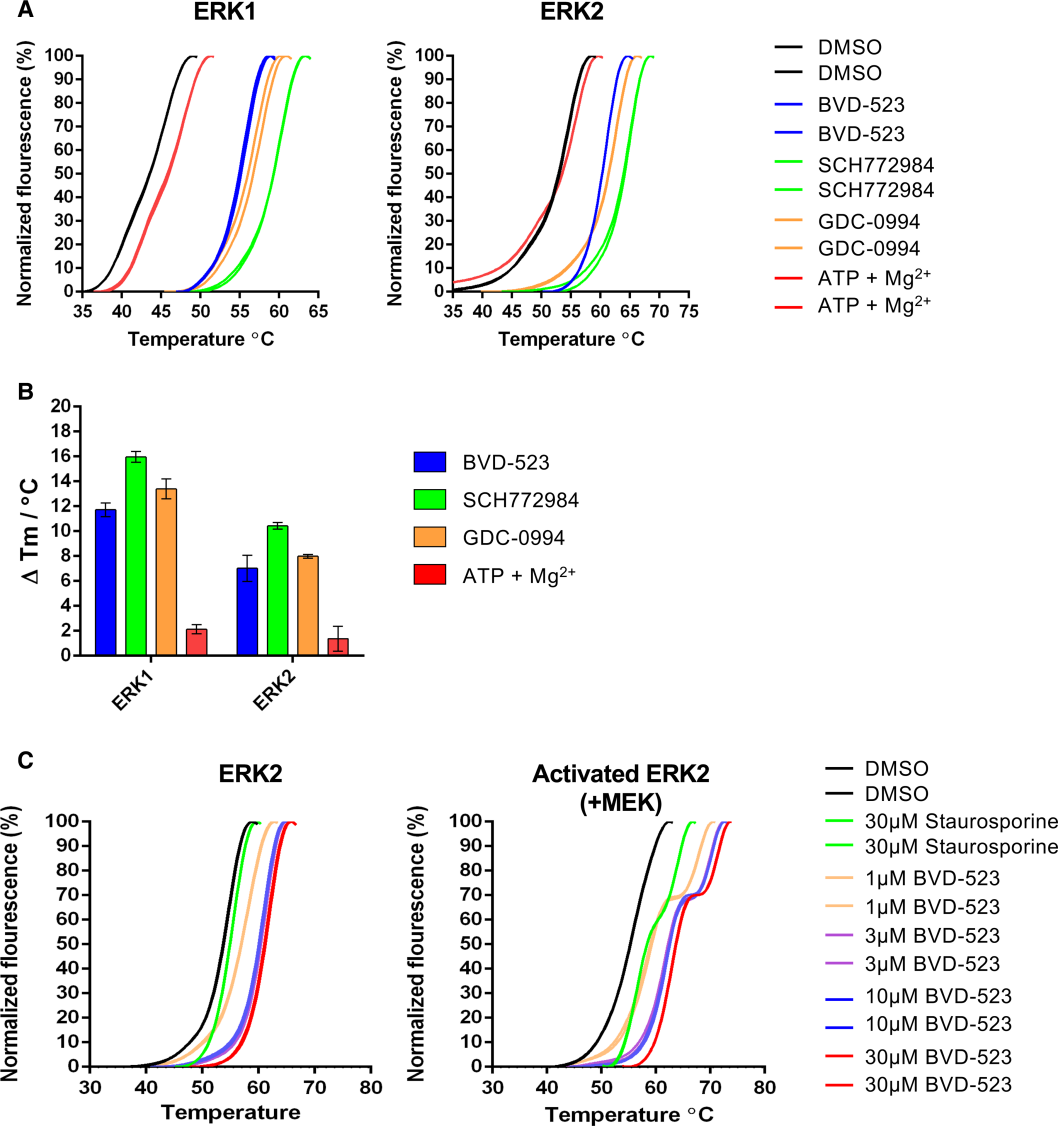
ERK1/2 inhibitors stabilise purified ERK1 and ERK2 *in vitro*. (**A**) Thermal denaturation profiles of purified ERK1 or ERK2 unfolding in the presence of 30 µM of the indicated inhibitor compound. Representative unfolding profiles from two independent experiments are shown. (**B**) Thermal shifts induced in the presence of 30 µM ERK1/2 inhibitor or 1 mM ATP: 10 mM Mg^2+^ ions. Data are means ± SD of *N* = 4 independent experiments. (**C**) Thermal denaturation profiles of ERK2 or MEK-activated ERK2 observed in the presence of the indicated concentration of BVD-523 or the type I ATP-competitive kinase inhibitor staurosporine (30 µM). Representative unfolding profiles from two independent experiments are shown.

We considered the possibility that ERKi binding might directly destabilise ERK2 leading to its degradation. To assess this we employed differential scanning fluorimetry (DSF) [24] to monitor ERK1/2 thermal denaturation profiles in the presence of ERKi. In this assay, reduced melting temperature (*T*_m_) is indicative of destabilisation, whereas increased *T*_m_ is indicative of stabilisation [25]. Initially, we noted that inactive (non-phosphorylated) ERK1 (2–379) and ERK2 (2–360) purified from *E. coli* had markedly different *T*_m_ values (44.2 ± 0.2°C and 54.1 ± 0.2°C, respectively), indicating that the purified ERK2 protein was much more thermostable than ERK1 (Figure 3A). We next examined the effect of BVD-523, SCH772984 and GDC-0994 on the stability ERK1 and ERK2; these three compounds were chosen based upon their rank order for driving loss of ERK2 in cells (BVD-523 > SCH772984 > GDC-0994). All three ERKi caused an increase in *T*_m_ for both ERK1 and ERK2 (Figure 3A,B) indicating ERKi-dependent stabilisation of each kinase *in vitro*. SCH772984 consistently gave the greatest stabilisation (Figure 3A,B), perhaps related to its unique binding mode [17]. Focusing more closely on ERK2, we found that BVD-523 also stabilised ERK2 that was activated by incubation with activated MEK1 and ATP and then re-purified (Figure 3C). Interestingly, for activated ERK2, we also observed a pronounced ‘shoulder’ on the melt curve at all doses of BVD-523 that was absent for non-MEK1-phosphorylated, inactive ERK2 examined under the same experimental conditions; a more subtle shoulder was also observed for the non-selective kinase inhibitor staurosporine. Whether this reflects variable binding and/or stabilisation of BVD-523 to two populations (e.g. a mixture of non-phosphorylated and phosphorylated) ERK2 is worthy of future investigation. Regardless, these biochemical data reveal that ERKi binding does not cause thermal destabilisation or denaturation of purified active or inactive ERK2 *in vitro*, which might otherwise account for the dramatic loss of ERK2 protein in ERKi-treated cells.

### ERKi promote the proteasome-dependent turnover of ERK2

**Figure 4.**
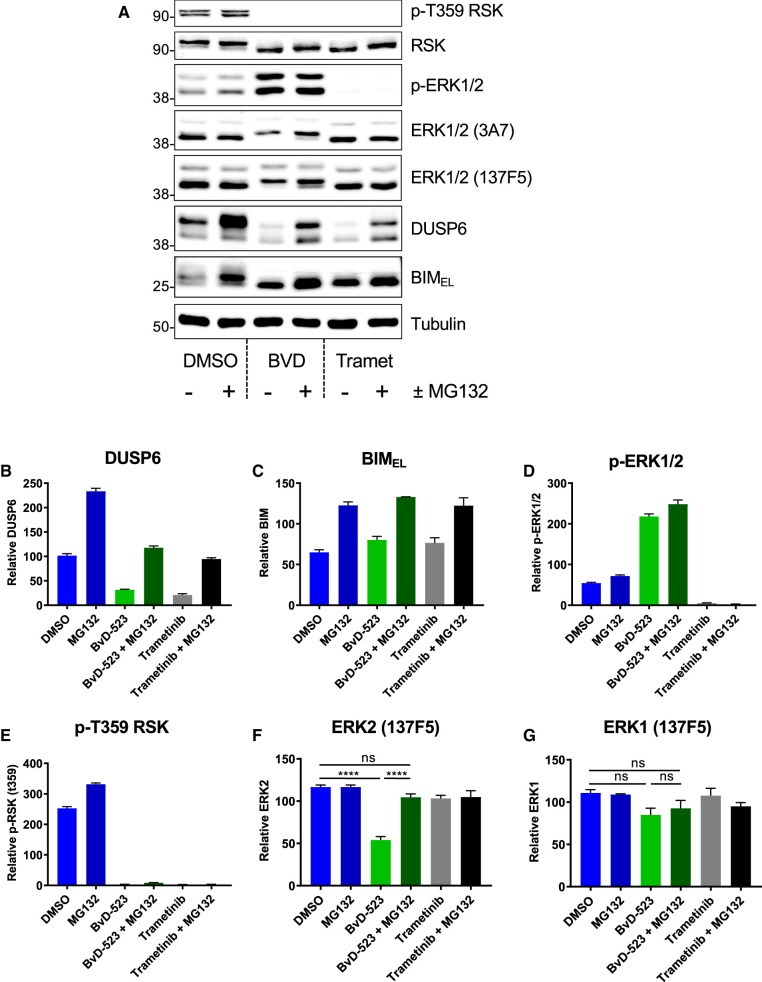
The ERK inhibitor BVD-523 promotes the proteasome-dependent turnover of ERK2. (**A**) HCT116 cells were exposed to 0.1% DMSO (control), BVD523 (3 µM) or Trametinib (100 nM) alone or plus (10 µM) MG132 for 24 h. Whole cell lysates were subjected to SDS–PAGE and immunoblotting with the indicated antibodies. Molecular masses in kDa are indicated on the left-hand side. (**B**–**G**) Results of Li-Cor quantitative western blotting for (**B**) DUSP6, (**C**) BIM, (**D**) p-ERK1/2 (pThr202/pTyr204-ERK1/2), (**E**) p-Thr359 p90RSK, (**F**) ERK2 (with ERK1/2 clone 137F5) or (**G**) ERK1 (with ERK1/2 clone 137F5). Results are the mean normalised blot quantification ± SEM, *n* = 3 experiments *P*-values *P* < 0.05 **, *P* < 0.01 *** *P* < 0.001 **** using one-way analysis of variance with Tukey's multiple comparison test.

To investigate the mechanism underlying the loss of ERK2 in cells we used MG132, an inhibitor of the 20S/26S proteasome (Figure 4). These experiments employed an 8 h treatment with BVD-523 ± MG132 to limit the toxicity of prolonged MG132 exposure. We monitored expression of DUSP6 and BIM_EL_ to confirm the efficacy of MG132 since they are degraded by the proteasome; indeed, both proteins increased in abundance when cells were treated with MG132 (Figure 4A–C). MG132 treatment caused only modest changes in p-ERK1/2 or p-T359-RSK (Figure 4A,D,E). In contrast, although MG132 treatment had no effect on the basal level of ERK1/2 in untreated cells, it completely reversed the loss of ERK2 observed in response to BVD-523 (Figure 4A,F). ERK1 abundance was not influenced by BVD-523 or MG132 (Figure 4A,G). Thus, ERKi promote the proteasome-dependent turnover of ERK2, but not ERK1, in cells.

### ERKi promotes the poly-ubiquitylation of ERK2

**Figure 5.**
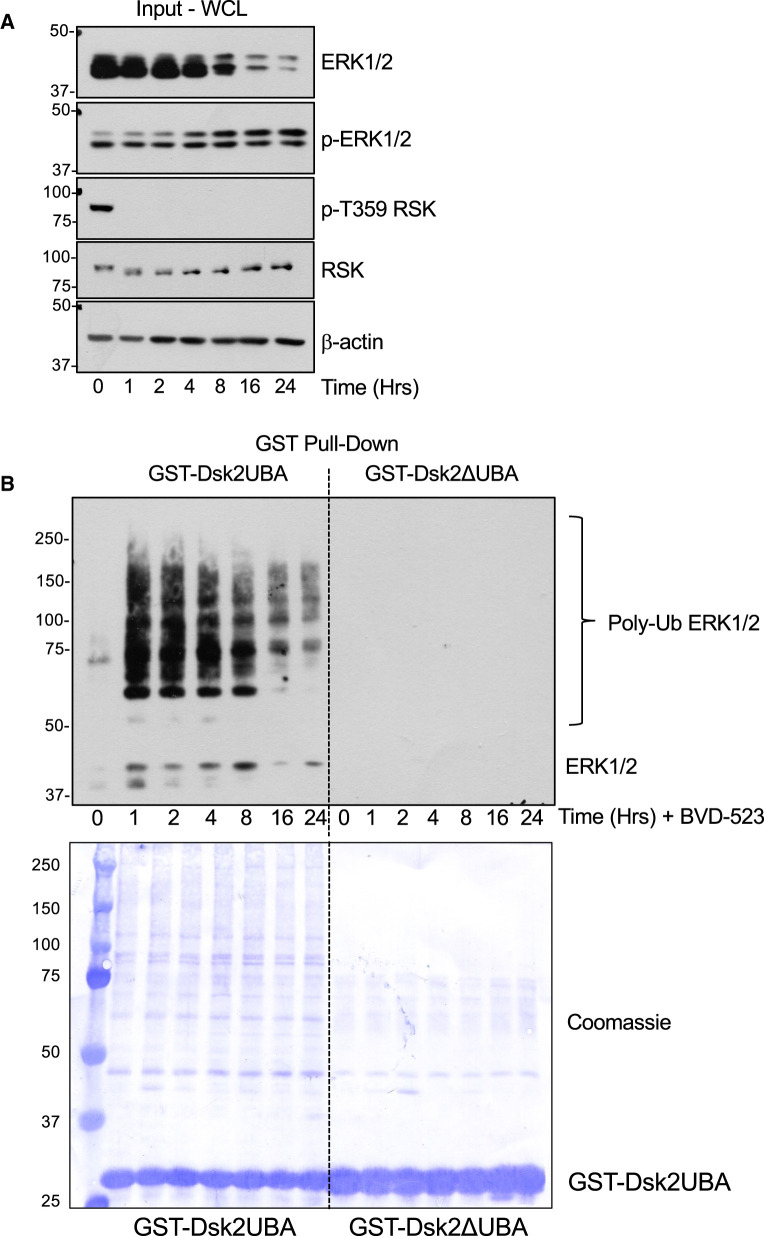
The ERK1/2 inhibitor BVD-523 promotes the polyubiquitylation of ERK1/2. (**A**) HCT116 cells were treated with 3 µM BVD-523 for the indicated times. Cells were lysed in TG-lysis buffer and a portion retained as input whole cell lysate (WCL) fractionated by SDS–PAGE and immunoblotted with the indicated antibodies. (**B**) Equal quantities of input lysates were incubated with GST-Dsk2 or GST-Dsk2ΔUBA beads. Proteins were eluted from the beads after washing by boiling in 1× SB, fractionated by SDS–PAGE and immunoblotted with the ERK1/2 antibody 137F5. The PVDF filters used were then stained with Coomassie blue to visualise precipitated protein and the relevant GST-Dsk2 fusion protein. Molecular masses in kDa are indicated on the left-hand side. Results are shown from a single experiment; identical results were observed in three experiments.

Many cellular proteins can be targeted to the 26S proteasome by poly-ubiquitylation; however, intrinsically disordered proteins may also be degraded by 20S proteasomes without prior ubiquitylation [26,27]. To determine if ERKi treatment promoted the polyubiquitylation of ERK2 we performed a ‘pull-down’ assay using the immobilised UBA domain of the yeast proteasome subunit Dsk2 as an affinity probe; this protein binds polyubiquitylated proteins with high affinity and exhibits some selectivity for K48-linked ubiquitin chains over K63-linked Ubiquitin chains [28]. We treated HCT116 cells with BVD-523 over a time course of 1–24 h and performed a pull down from whole cell extracts using GST-Dsk2UBA beads; as control we also performed pull-downs with GST-Dsk2ΔUBA, which contains two mutations (M342R and F344A) in the UBA domain that impair ubiquitin binding [28]. Analysis of whole cell lysates (Figure 5A, Input WCL) confirmed a striking reduction in ERK2 abundance from 4 h onwards after BVD-523 exposure. Wild-type GST-Dsk2UBA beads captured polyubiquitylated forms of ERK1/2 from lysates of BVD-523-treated cells; this was maximal at 1–4 h and declined from 8 to 24 h, likely as a consequence of proteasome-dependent destruction of ubiquitylated ERK2 protein (Figure 5B, GST-Dsk2 UBA Pull Downs). In contrast no polyubiquitylated ERK1/2 was captured by GST-Dsk2ΔUBA (Figure 5B) despite Coomassie blue staining of PVDF membranes revealing that slightly more mutant GST-Dsk2ΔUBA was employed in the pull-down than wild type GST-Dsk2UBA (Figure 5B). The rapid appearance of polyubiquitylated ERK1/2 1 h after BVD-523 treatment clearly preceded the slower loss of ERK2, as would be expected if ubiquitylation was induced as a consequence of drug binding and served as a signal for degradation. Importantly, enhanced poly-ubiquitylation of ERK1/2 was also observed in response to treatment with other ERKi, including SCH772984 and Compound 27, but not in response to the MEKis Selumetinib or Trametinib (Supplementary Figure S5).

**Figure 6.**
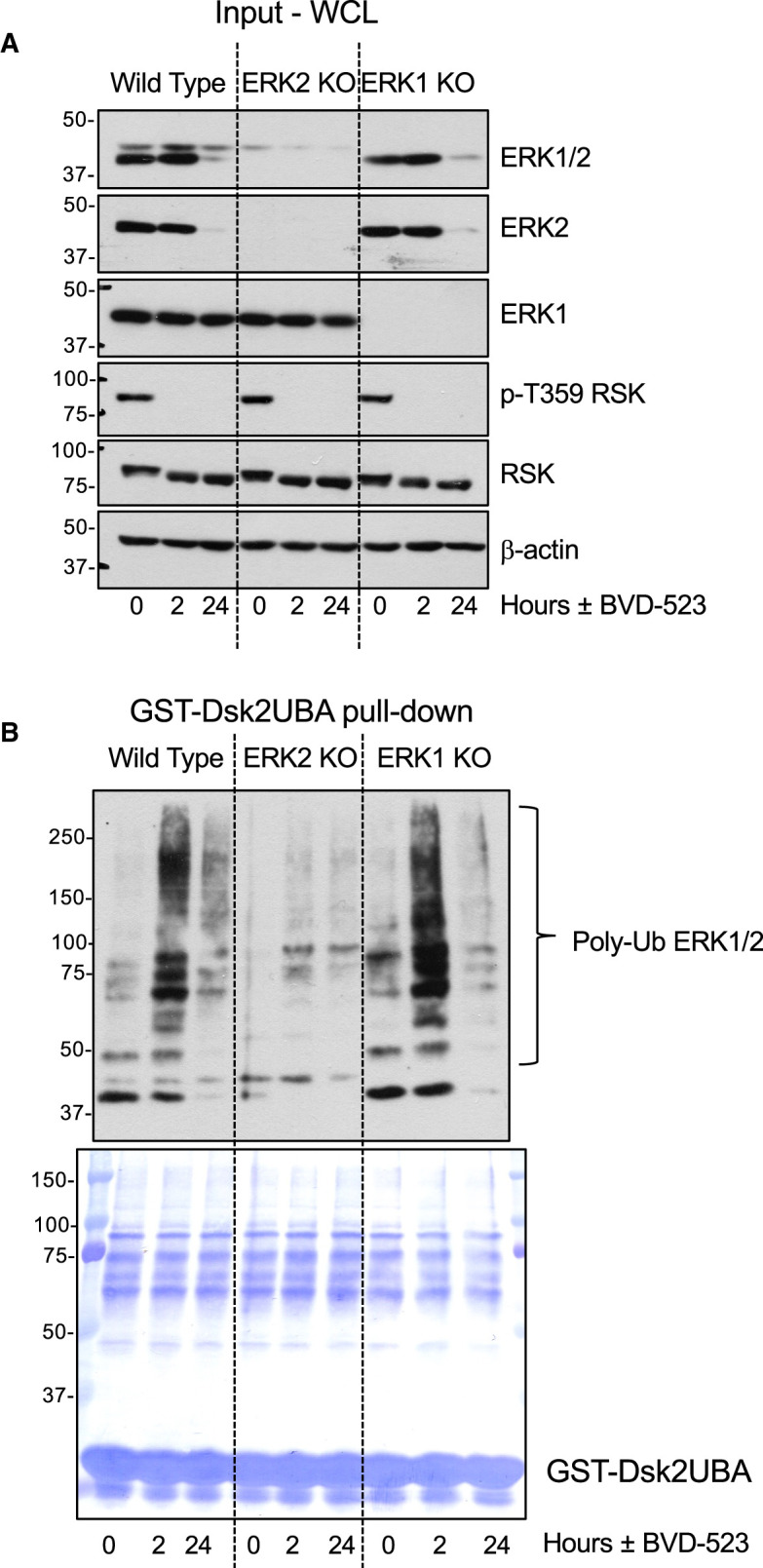
The ERK1/2 inhibitor BVD-523 selectively promotes the polyubiquitylation of ERK2 with little, if any, polyubiquitylation of ERK1. (**A**) Wild type, ERK2 KO or ERK1 KO HCT116 cells were treated with 3 µM BVD-523 for 2 or 24 h. Cells were lysed in TG-lysis buffer and a portion retained as input whole cell lysate (Input WCL) fractionated by SDS–PAGE and immunoblotted with the indicated antibodies. (**B**) Equal quantities of input lysates in A were incubated with GST-Dsk2UBA beads. Proteins were eluted from the beads after washing by boiling in 1× SB, fractionated by SDS–PAGE and immunoblotted with the ERK1/2 antibody 137F5. The PVDF filters used were then stained with Coomassie blue to visualise precipitated protein and the GST-Dsk2UBA fusion protein. Molecular masses in kDa are indicated on the left-hand side. Results are shown from a single experiment; identical results were observed in three experiments.

We have found the pan-ERK1/2 antibody to be superior for western blotting of lysates and GST-Dsk2UBA pull downs so we could only infer that the poly-ubiquitylated ERK1/2 signal was ERK2 because ERK1 exhibited little or no turnover. To confirm this we performed GST-Dsk2UBA pull downs on lysates from wild type HCT116 cells treated with BVD-523 for 2 or 24 h and compared with ERK2 KO or ERK1 KO cells prepared by CRISPR-Cas9 gene editing (Figure 6A,B). BVD-523-induced polyubiquitylation of ERK1/2 detected with the pan-ERK1/2 antibody in WT cells was greatly reduced in ERK2 KO cells but proceeded normally in ERK1 KO cells that retained ERK2 expression. The input lysates confirmed the knock-out of ERK1 and ERK2 in the relevant cell lines and the loss of ERK2 but not ERK1 upon BVD-523 treatment (Figure 6A). Taken together, our results demonstrate that ERKi selectively drive the polyubiquitylation and proteasomal degradation of ERK2 with little, if any, effect on ERK1.

### ERKi drives the cullin-dependent turnover of ERK2

Ubiquitylation and proteasomal degradation of proteins requires the co-ordinated action of E1, E2 and E3 ubiquitin ligase complexes. The majority of the >600 mammalian E3 ligases belong to the ‘really interesting new gene’ (RING) family and include over 400 cullin-RING E3 ligases (CRLs) which are thought to be responsible for ∼20% of all ubiquitylation in cells [29,30]. The core CRL consists of: one of seven cullin proteins that serve as scaffolds; a RING finger protein that binds to an E2 ubiquitin conjugating enzyme; a substrate receptor that recognises the target protein (for example an F-box protein), and an adaptor protein that bridges the substrate receptor to the cullin [29,30]. Efficient substrate ubiquitylation by CRLs requires the covalent modification of a conserved lysine in the C-terminal domain of all cullins by a ubiquitin-like molecule, NEDD8 [31,32]. Like ubiquitylation, this is catalysed by the sequential action of an E1 NEDD8-activating enzyme (NAE) and an E2 NEDD8-conjugating enzyme (UBC12). To assess if cullin activity was required for ERK2 turnover we employed MLN4924 [33], a potent and selective NAE/E1 inhibitor, and a dominant negative mutant of UBC12 (DN-UBC12) [34] that we have previously used [28].

**Figure 7.**
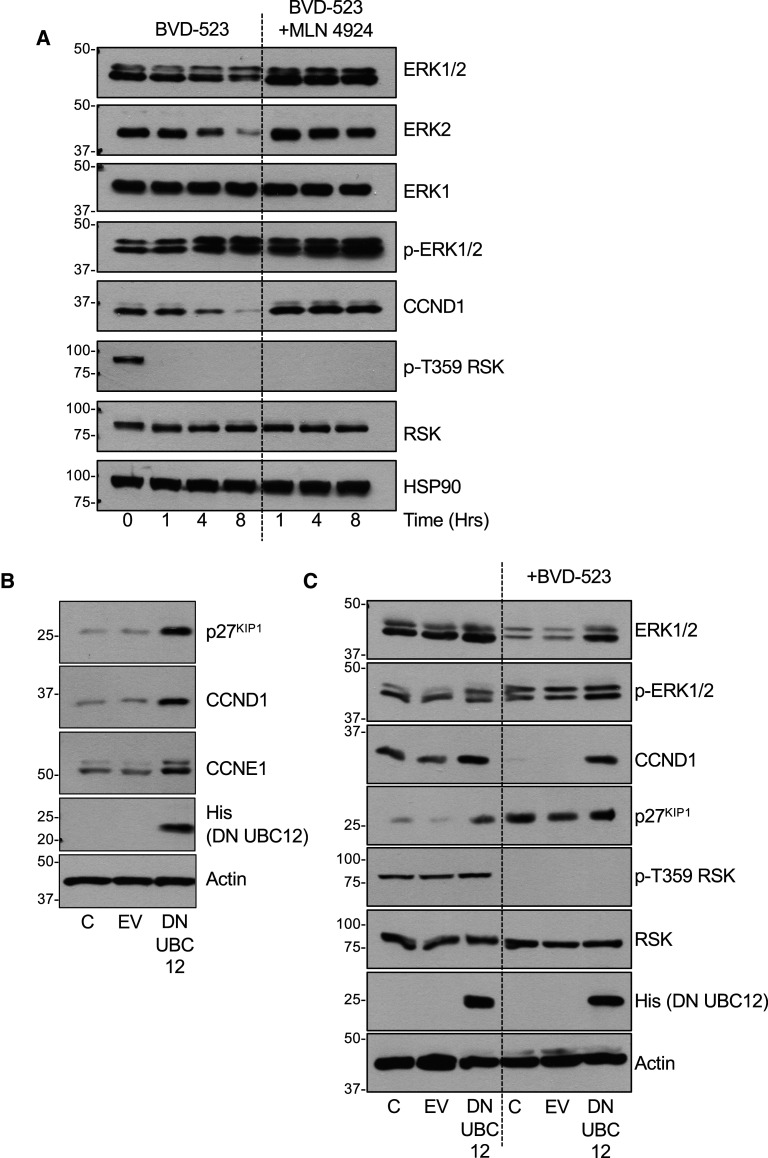
ERKi-driven ERK2 turnover depends a cullin family E3 ubiquitin ligase. (**A**) HCT116 cells were treated with 3 µM BVD-523 with or without 300 nM MLN4924 for the indicated times. Cells were lysed in TG-lysis buffer, fractionated by SDS–PAGE and immunoblotted with the indicated antibodies. (**B**) HCT116 cells were transiently transfected with empty vector (EV) or His-tagged dominant negative UBC12 (DNUBC12). After 24 h cells were lysed in TG-lysis buffer, fractionated by SDS–PAGE and immunoblotted with the indicated antibodies. (**C**) HCT116 cells were transiently transfected with empty vector (EV) or His-tagged dominant negative UBC12 (DNUBC12). After 24 h cells were treated with DMSO or 3 µM BVD-523 for 24 hours. Cells were lysed in TG-lysis buffer, fractionated by SDS–PAGE and immunoblotted with the indicated antibodies. Molecular masses in kDa are indicated on the left-hand side. Results are shown from a single experiment; identical results were observed in three experiments.

We first validated the efficacy of MLN4924 by showing that it blocked the BVD-523-driven turnover of cyclin D1 (CCND1), a known cullin substrate [35] in HCT116 cells (Figure 7A). In the same experiment BVD-523 promoted the turnover of ERK2 (but not ERK1) and this was completely prevented by MLN4924 (Figure 7A). Furthermore, MLN4924 not only prevented BVD-523-driven ERK2 turnover at 8 h but also strongly inhibited poly-ubiquitylation of ERK2 at 1, 4 and 8 h (Supplementary Figure S6). Next, we validated DN-UBC12 by transient expression in HCT116 cells; this was sufficient to increase the basal abundance of three known cullin substrates: CCND1 [35], CCNE1 [36] and p27^KIP1^ [36] (Figure 7B). BVD-523 again stimulated turnover of CCND1 and ERK2 (but not ERK1), and in both cases this was reversed by transient expression of DN-UBC12 (Figure 7C). Thus, both pharmacological and genetic inhibition of cullin function prevented the BVD-523-dependent poly-ubiquitylation and turnover of ERK2, indicating a requirement for a cullin-RING E3 ligase complex.

### Loss of ERK2 T-E-Y dual phosphorylation prevents ERKi-driven ERK2 turnover

**Figure 8.**
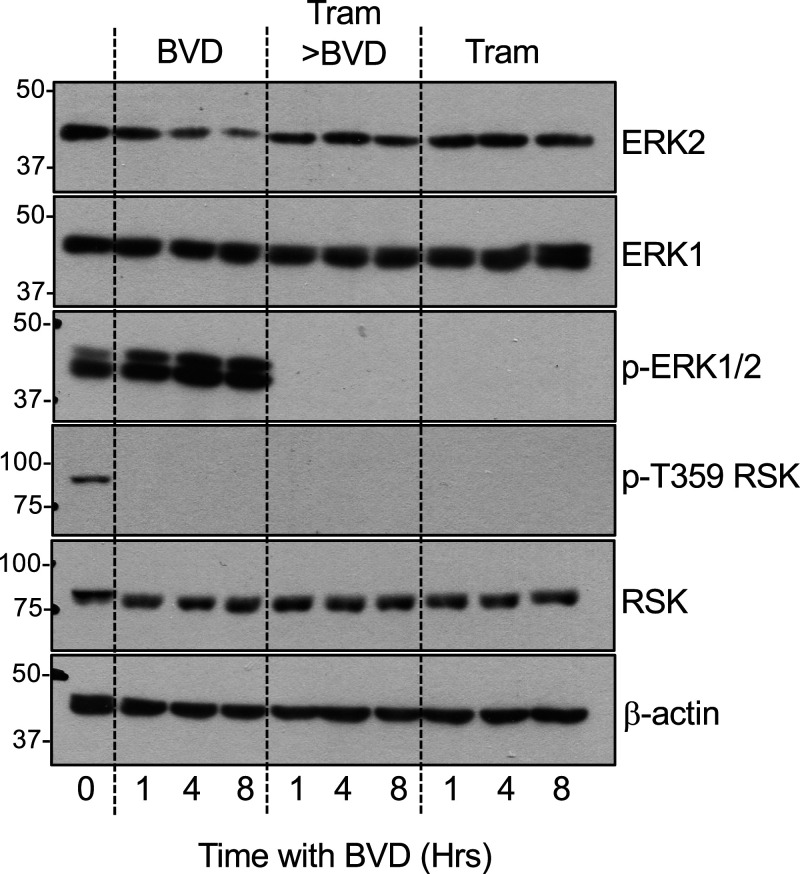
Inhibition of MEK1/2 prevents BVD-523-driven ERK2 turnover. HCT116 cells were treated with DMSO (Vehicle), 100 nM Trametinib for 24 h followed by 3 µM BVD-523 with or without 100 nM Trametinib for the times indicated. Cells were then lysed in TG-lysis buffer, fractionated by SDS–PAGE and immunoblotted with the indicated antibodies. Molecular masses in kDa are indicated on the left-hand side. Results are shown from a single experiment; identical results were observed in three experiments.

The failure of MEKis (Selumetinib, Trametinib) to drive ERK2 turnover suggested that ERK2 degradation was a consequence of ERKi binding but not a consequence of loss of MEK-catalysed pT-E-pY dual phosphorylation. However, in unstimulated cells inactive ERK2 binds directly with MEK1 or MEK2 [4,37,38]. MEK1/2-catalysed dual pT-E-pY phosphorylation not only activates ERK2, but also releases it from MEK1/2 so that it can engage with substrates outside and inside the nucleus. Based on this model, we pre-treated cells with Trametinib to fully inhibit pT-E-pY dual phosphorylation of ERK1/2; this inhibited the BVD-523-induced turnover of ERK2, whereas Trametinib treatment alone had no effect on ERK2 stability, as previously established (Figure 8). A trivial explanation for this, that BVD-523 exhibits differential binding to non-phospho versus phospho-ERK2, as is seen with MEKi binding to MEK [39], seems unlikely since BVD-523 bound very effectively to both phospho- and non-phospho-ERK2 (Figure 3C). So, whilst increase (by FBS) or loss (by MEKi) of pT-E-pY alone has no effect on ERK2 stability *per se* (Figure 1) by adding a space after *per se*, loss of pT-E-pY dual phosphorylation (which prevents ERK1/2 dissociation from MEK1/2) also prevents the turnover of ERK2 driven by ERKi binding. Thus, non-phosphorylated ERK2 that is complexed with MEK1/2 may be protected from ERKi driven turnover. This suggests that it is ERKi binding to p-T-E-pY ERK2, which is not bound to MEK, that is sensitive to ERKi-driven turnover. Whilst dmERKi such as Compund 27 or SCH772984 also reduce rebound pT-E-pY phosphorylation they were still able to drive ERK2 degradation; this is likely because they reduce, but do not abolish, pT-E-pY phosphorylation as is seen with Trametinib (Figures 2A,B, 8).

## Discussion

The discovery that intrinsic and acquired resistance to BRAFi and/or allosteric MEKi usually arises through reactivation of ERK1/2 [11,12,15] has fuelled the development of a range of catalytic or dual-mechanism small molecule ERK inhibitors (ERKi), many of which are undergoing clinical evaluation [16,17,23]. We previously noted that ERKi treatment appeared to cause a reduction in ERK1/2 abundance [17]. In this study, we show that both catERKi and dmERKi, are able to selectively promote the proteasome-dependent degradation of ERK2. Degradation of ERK2 was most striking with BVD-523 but was readily apparent with eight of nine different ERKi that we tested. Differences in efficacy do not seem to stratify with catERKi or dmERKi and likely reflect other properties of the inhibitors such as ‘on’ and ‘off’ rates that determine occupancy time. Clearly further work is required in this area guided by the properties of BVD-523. Using BVD-523 as the exemplar, any minor changes in ERK1 abundance were modest and variable across experiments whereas the loss of ERK2 was striking and consistent across all experiments and all cell lines.

Interestingly, even a striking loss of ERK2 had little impact on the level of dual-phosphorylated pT-E-pY ERK2 (p-ERK2); p-ERK2 levels were either sustained or increased slightly as ERK2 levels declined. This reflects two key aspects of the ERK1/2 pathway. First, loss of ERK1/2 activity prevents feedback inhibition of RAF, resulting in de-repression and activation of RAF, increased MEK1/2 activity and phosphorylation of ERK1/2; this homeostatic mechanism allows the pathway to adapt to ERK1/2 inhibition. Second, this robustness is facilitated by the fact that just a small fraction of the total ERK1 and ERK2 pool is in the pT-E-pY phosphorylated active state at any one time. For example, in HCT116 cells harbouring a KRAS^G13D^ mutation just 5% of the ERK1 and ERK2 pool is in the active pT-E-pY phosphorylated state [40]. So there is a very large ‘spare capacity’ for further ERK2 activation and this is seen in the maintenance (or even small increase in) p-ERK2 levels in response to BVD-523 treatment. The same applies for ERK1 but in several experiments we noted that the increase p-ERK1 was more apparent than the increase in p-ERK2 over prolonged treatment times. For example, this was seen for BVD523 (Figure 1A,C, 5A, 6A, Supplementary Figures S1B, S2A), VTX-11e (Supplementary Figure S1A), GDC-0994 (Supplementary Figure S1B) and AZ6197 (Supplementary Figure S1B). Notably, it has been suggested that ERK1 and ERK2 normally compete for phosphorylation and activation by MEK1/2, with ERK2 phosphorylation preferred simply because it is more abundant [41]. Thus, the loss of total ERK2 following ERKi may limit the magnitude of the increase in p-ERK2 but facilitate the increase in p-ERK1. Again, this exemplifies how robust the ERK1/2 pathway is to drug-induced interventions.

What might be the significance of ERK2 degradation? Whilst ERK1/2 are best known for their ability to phosphorylate hundreds of substrates, including many transcription factors, they have also been proposed to have catalysis-independent functions including interactions with topoisomerase II [18], PARP1 [19], and DUSP6 [20]. Binding of nuclear ERK1/2 to lamin A displaces the retinoblastoma (RB) protein, facilitating its phosphorylation by cyclin-dependent kinases to promote cell-cycle entry [21]. In addition, ERK2 specifically has been proposed to act as a transcriptional repressor of IFN-responsive genes by directly binding to their promoter regions [22]. In this context the ability of catERKi such as BVD-523 to increase pT-E-pY dual phosphorylated but inactive ERK1/2 in the nucleus [17] may allow such catalysis-independent ERK1/2 signalling to persist. Thus, degradation rather than simple inhibition might be an advantage in terminating all aspects of ERK2 signalling; this may also be a more desirable property in therapeutic terms. Nonetheless, acquired resistance to ERKi that drive ERK2 degradation takes place [17] and future studies should seek to understand the mechanisms, whether ERKi-dependent ERK2 degradation delays the onset of acquired resistance to BRAFi and/or MEKi and whether ERK1 degradation is also required for therapeutic effects.

Recent years have seen an explosion in the field of targeted protein degradation (TPD) technologies [42], best exemplified by PROteolysis TArgeting Chimeras (PROTACs), which are heterobifunctional small molecules that combine a specific ligand for a target protein of interest (POI) and an E3 ubiquitin ligase (E3) binding motif, thereby driving the ubiquitylation and proteasome-dependent degradation of the POI [43]. TPD strategies not only ablate enzymatic functions but all other functional domains; this is relevant to the many protein kinases that possess non-catalytic functions (scaffolding, allosteric regulation, etc) mediated by protein–protein interactions (PPIs) that contribute to disease phenotypes. For example, in cells with wild type BRAF, BRAFi binding to one BRAF protomer in a BRAF:BRAF or BRAF:CRAF dimer results in allosteric activation of the non-drug-bound dimer partner resulting in paradoxical activation of ERK1/2 signalling and adventitious tumour growth in non-melanoma tissue [44]. In comparison with wild type p110α PI3K, an oncogenic mutant exhibits enhanced protein–protein interactions, notably with IRS1, that contribute to its oncogenic properties [45] and which are not targeted by traditional PI3K inhibitors. Finally, ERK5 inhibitors effectively inhibit ERK5 kinase activity but cause the paradoxical activation of the ERK5 C-terminal transactivation domain [46,47]. In such scenarios, PROTACs that target catalytic and non-catalytic functions of a protein kinase by TPD may provide a greater and more sustained therapeutic effect. Indeed, an ERK5 PROTAC has recently been described [48]. Attempts have been made to develop targeted degraders for ERK1/2 including CLIPTAC, a modified PROTAC in which a covalent ERK1/2 inhibitor is ‘armed’ with a reactive group that undergoes a click reaction with an E3 ligase ligand, thereby assembling an ERK1/2 PROTAC in cells [49]. This approach has proved successful for rapid degradation of ERK1/2, and has been adapted to allow imaging of endogenous ERK1/2 bound to a covalent ERKi in cells [50]. Whilst CLIPTAC is an elegant advance on the PROTAC method, our results suggest that a PROTAC-based approach to degrading ERK1/2 may not be necessary since both catERKi and dmERKi are able to drive the ubiquitylation and degradation of the most abundant ERK2 isoform.

An increasing number of protein kinase inhibitors have been found serendipitously to promote degradation of their cognate targets, including monovalent inhibitors of HER2, LRRK2 CHK1, BTK, MELK, SK1, c-KIT and JAK2/3 [51]. In the majority of cases these ‘monovalent kinase degraders’ drive proteasome-dependent degradation, though in some cases lysosomal degradation is also implicated. Some EGFR family kinase inhibitors can also fortuitously drive ‘off-target’ degradation of pseudokinases such as Tribbles 2 (TRIB2), where compound effects are independent of catalytic activity modulation, and instead drive conformational switching that leads to auto-degradation through the proteasome [25]. Our results suggest that chemical ERKi may also act as monovalent kinase degraders of ERK2 by driving the assembly of a ubiquitin-mediated signalling module that leads to its rapid degradation.

The precise mechanism that initiates ERKi-driven ERK1/2 turnover is currently unclear. Whilst our results clearly show that ERKi promote cullin-dependent ubiquitylation and proteasomal degradation of ERK2 there remain a number of outstanding questions. First, what is the ‘signal’ for ERK2 ubiquitylation? Increases or decreases in pT-E-pY dual phosphorylation alone have no effect on ERK2 abundance but ERK2 turnover is seen with eight different ERKi (both catERKi and dmERKi) and ‘tracks’ with readouts of ERKi binding and inhibition (loss of RSK phosphorylation, up-regulation of BIM) suggesting that ERKi binding is the key initiator. However, this is not sufficient since pre-treatment with trametinib (a MEKi) to block pT-E-pY dual phosphorylation inhibits ERKi-induced ERK2 turnover. Rather, it suggests that it is ERKi binding to pT-E-pY dual phosphorylated ERK2 that signals its turnover. Alternatively, since loss of pT-E-pY dual phosphorylation also prevents dissociation of ERK2 from MEK [4,37,38] it may be that it is p-ERK2, which is not bound to MEK, that is sensitive to ERKi-driven turnover. These alternatives are not mutually exclusive; for example, it may be that ERKi binding to the ‘free and phosphorylated’ form of ERK2 exposes an otherwise cryptic degron for a cullin-based E3 ligase. The persistence of p-ERK2 at 16–24 h when ERK2 is being degraded might contradict the suggestion that pT-E-pY phosphorylation is required for the turnover. However, given the ‘spare capacity’ for ERK1/2 activation [40], it is important to appreciate that at any one timepoint there will be ERK2 molecules that are (a) not phosphorylated, (b) phosphorylated but have not yet dissociated from MEK1/2, (c) phosphorylated and dissociated but have not yet been ubiquitylated or (d) phosphorylated and ubiquitylated but have not yet been degraded by the proteasome. Regardless, our results suggest that in cells in which the pathway is not stimulated, ERK2 may be protected from ERKi-driven turnover by binding to MEK1/2; conceivably, binding to other pathway components (scaffolds, substrates) might also protect ERK2 from ERKi-driven turnover. Expression of TEY-to-AEF mutants of ERK2 may go some way to address this in the future but since we do not know if N- or C-terminal degrons are involved this may require re-expression of untagged ERK2 proteins in ERK2 null cells. A second question is whether ERKi are actually acting as molecular glues, small molecules that bring together two proteins that do not normally interact. Perhaps ERKi bridge an interaction between ERK2 and an E3 ligase? Or ERKi binding may change the molecular surface of ERK2, exposing a degron or specific lysine residues that can be targeted by an E3 ligase? A remaining key question is why ERK2 is degraded but ERK1 is not. ERK1 and ERK2 are activated and inactivated in the same way and there are few robust or reproduced examples of different substrates, scaffolds or biological functions. ERK1 and ERK2 exhibit 85% sequence identity and the only notable difference is a 17 amino acid insert towards the N-terminus of ERK1, that is implicated in slower nuclear entry [52]. Furthermore, studies suggest that any differences in ERK1 or ERK2 biology largely reflect their relative abundance [53] since embryonic lethality due to knockout of the more abundant *erk2* can be rescued by expression of *erk1* from a strong ubiquitous β-actin promoter [54]. ERK1:ERK2 fragment switch mutants may shed additional light on the key sequence determinants that promote rapid ERK2 turnover. Finally, critical insights will come from identifying which lysine(s) in ERK2 are ubiquitylated, the precise nature of the ubiquitin chain linkages and which of the ∼400 cullin-RING E3 ligases is/are responsible.

In summary, we have shown that eight chemically distinct ERKi share the ability to drive the ubiquitylation and proteasomal degradation of ERK2, the most abundant of the two canonical ERK isoforms. This finding may point to further development of new classes of ERKi in which ERK2 degradation and ERK pathway inhibition is fine-tuned, perhaps as enhanced first line monotherapies or in approaches to overcome acquired resistance to MEKi or BRAFi. In the case of ERK2 degradation, this might be especially relevant in melanoma, an ERK1/2-driven disease which exhibits a strong dependency on ERK2, but not ERK1 (Cancer Dependency Map https://depmap.org/portal/). In addition, understanding the degradation of ERK2 by ERKi may allow similar targeting of ERK1 and might provide a useful set of tools and reagents to better understand proposed kinase- or catalysis-independent functions of ERK1 or ERK2. Finally, our results also argue that use of ‘total ERK1/2’ or ‘total ERK2’ as a loading control for immunoblots should cease, especially in experiments employing ERKi.

## Materials and methods

### Materials

BVD-523, GDC-0994 and SCH72984 were purchased from Chem Express and LY3214996, VTX-11e and FR180204 from Selleck. AZ7370 and AZ6197 were provided by AstraZeneca. Compound 27, a precursor of ASTX029 [23] was provided by Astex Pharmaceuticals. BVD-523, GDC-0994, SCH72984 LY3214996 and Compound 27 were previously characterised for their effects on ERK1/2 signalling [17]. Selumetinib and Trametinib were both purchased from Selleck Chemicals. MG132 was purchased from Sigma. GSH-conjugated sepharose beads were purchased from GE healthcare. All other standard reagents were purchased from Sigma. Dulbecco's Modified Eagle's Medium (DMEM), foetal bovine serum (FBS), Penicillin/Streptomycin and glutamine were all purchased from Life Technologies. Enhanced chemiluminescence reagents (ECL) were purchased from GE Healthcare, horseradish peroxidase (HRP) conjugated secondary antibodies from Bio-Rad, the Dylight^TM^680 and Dylight^TM^800 conjugated secondary antibodies were from Cell Signalling Technology. Antibodies for ERK1/2 (clones 3A7 (9107) and 137F5 (4695)), pThr202/Tyr204 ERK1/2 (4370), ERK2 (9108), p90-RSK (9355), pThr359 p90RSK (8753), MEK 1/2 (9122), pSer217/Ser221 MEK 1/2 (9154), and Cyclin D1 (2798) were purchased from Cell Signalling Technology. The BIM antibody (AB17003) was purchased from Merck-Millipore and the ERK1 (ab32537) and DUSP6 antibodies from Abcam (ab76310). The α-tubulin (T9026) and β-actin (A5441) antibodies were purchased from Sigma. HT29, SW480 and Colo205 cells were all purchased from ATCC. HCT116 cells obtained from Bert Vogelstein (Johns Hopkins University, Baltimore, MD, U.S.A.) and A375 cells were obtained from Richard Marais (Cancer Research U.K. Manchester Institute, Manchester, U.K.). For thermal stability assays, both unactivated and MKK1-activated human ERK1/2 (ERK1, amino acids 2–379 [DU1509] and ERK2, amino acids 2–360 [DU650]) were purchased from MRC PPUU reagents (University of Dundee). These were expressed in *E. coli* as N-terminally GST-tagged recombinant proteins and the GST-tag was removed prior to analysis.

## Methods

### Cell culture

Cells were grown in DMEM (HCT116, A375), Leibovitz's L-15 (SW480), McCoy's 5A (HT29) or RPMI1640 (COLO205) medium supplemented with 10% (v/v) FBS, penicillin (100 U/ml), streptomycin (100 mg/ml), and 2 mmol/L glutamine. Cells were incubated in a humidified incubator at 37°C and 5% (v/v) CO_2_. All cell lines were authenticated by short tandem repeat profiling. Cell lines were monitored regularly for mycoplasma infection.

### SDS–PAGE and western blotting

After inhibitor treatment, cells were lysed in ice-cold TG-lysis buffer, assayed for protein content and fractionated by SDS–PAGE as previously described [40]. Western blotting with HRP-conjugated secondary antibodies and ECL detection was performed as described previously [40]. To enable the quantification of western blots we employed fluorescently tagged secondary antibodies on a Li-Cor Odyssey Imaging System (LI-COR Biosciences) [17]. Briefly, membranes were cut to allow probing for multiple molecular weight proteins. Where appropriate, blots were probed with antibodies raised in different species, using multiple colours to detect proteins of similar molecular weight on the same membrane. If necessary, multiple independent blots were performed using the lysate from each experiment. For Li-Cor quantification the protein of interest was normalised to a tubulin loading control.

### Thermal stability assay by differential scanning fluorimetry

Thermal shift assays were performed using a StepOnePlus real-time polymerase chain reaction (PCR) machine (Life Technologies) with SYPRO Orange dye (Invitrogen) and thermal ramping (0.3°C step intervals between 25° and 94°C) in a 96 well-plate format. Purified ERK1 or ERK2 were assayed at a final concentration of 5 µM in 50 mM tris-HCl (pH 7.4) and 100 mM NaCl in the presence or absence of ATP and Mg^2+^ (1 mM and 10 mM, respectively) or the appropriate concentration of kinase inhibitor compound. The final concentration of DMSO was matched to inhibitor experiments, and never exceeded 4% (v/v), as described previously [24,55,56]. Normalised data were processed using the Boltzmann equation to generate sigmoidal denaturation curves, and average *T*_m_/Δ*T*_m_ values were calculated as previously described [57] using GraphPad Prism software.

### GST-Dsk2UBA pull down assay

GST-Dsk2UBA or the inactive M342R/F344A mutant (GST-Dsk2ΔUBA) were immobilised on GSH sepharose beads and used to pull down ubiquitylated ERK1/2 [28]. Briefly, cells were lysed in 1 ml ice cold TG lysis buffer, insoluble material separated by centrifugation (12 000 rpm, 10 min 4°C) and protein concentration estimated by Bradford assay. Lysates were pre-cleared by incubation with GSH sepharose with end-over-end mixing at 4°C for 30 min or boiled in sample buffer for Input whole cell lysate (Input WCL) control blots. After pre-clearing beads were pelleted and equal amounts of supernatant added to GST-Dsk2UBA or GST-Dsk2ΔUBA beads, which were then incubated for 2 h with end-over-end mixing at 4°C. The beads were washed three times with 1 ml ice cold TG-lysis buffer before being drained and boiled in 1× sample buffer. Samples were then fractionated by SDS–PAGE before immunoblotting.

### Data reproducibility

All individual experiments described have been repeated at least three times, and many have been repeated between four and six times. Initial observations of ERK2 degradation were made by Andrew Kidger. These were reproduced independently and then expanded on, with mechanistic analysis, by Kathryn Balmanno and Nejma Nassman. So key results have been repeated independently by three experimentalists.

## Data Availability

All relevant data are contained within the main article and its Supplementary Files.

## Abbreviations

CCND1: cyclin D1
CRLs: cullin-RING E3 ligases
DMEM: Dulbecco's Modified Eagle's Medium
ECL: enhanced chemiluminescence
ERKi: ERK1/2 inhibitors
HRP: horseradish peroxidase
NAE: NEDD8-activating enzyme
POI: protein of interest
PROTACs: PROteolysis TArgeting Chimeras
RING: really interesting new gene
TPD: targeted protein degradation
WCL: whole cell lysate
