# Supplementary material for: ERK1/2 inhibitors act as monovalent degraders inducing ubiquitylation and proteasome-dependent turnover of ERK2, but not ERK1
Source: Biochem J. 2023 May 4;480(9):587–605. doi: 10.1042/BCJ20220598 (PMC10212521; doi:10.1042/BCJ20220598)
Supplement: Supplementary Material [file BCJ-480-587-s1.pdf]

Legends for Supp Figures

Supp Figure 1. Three other catERKi, VTX-11e, AZ7370 and AZ6197 also promote the loss of ERK2, but not ERK1.

A. COLO205 cells were treated with the indicated doses of FR180204, VTX-11e, SCH772984 or AZ7370 for 72 hours. **B.** COLO205 cells were treated with the indicated doses of BVD-523, GDC-0994 or AZ6197 for 72 hours. For A & B whole cell lysates were fractionated by SDS-PAGE and immunoblotted with the indicated antibodies. Molecular masses in kDa are indicated on the left-hand side. Results are from a single experiment; identical results were obtained in 2 replicate experiments.

Supp Figure 2. catERKi and dmERKi drive the dose-dependent loss of ERK2 over the same dose range as they inhibit ERK1/2. COLO205 cells were treated the indicated doses of **A** BVD-523 or **B** SCH772984 for 72 hours. Cell lysates were fractionated by SDS-PAGE and immunoblotted with the indicated antibodies. Molecular masses in kDa are indicated on the left-hand side. Results are from a single experiment; identical results were obtained in 2 replicate experiments.

Supp Figure 3. catERKi and dmERKi promote the loss of ERK2, but not ERK1, in A375 melanoma cells and HT29 colorectal cancer cells. A375 cells (**A-C**) or HT29 cells (**D-F**) were treated with DMSO (D), the ERK inhibitors Compound 27 (27, 30nM A375, 300nM HT29), SCH772984 (S, 100nM A375, 1µM HT29), GDC-0994 (G, 3µM A375, 10µM HT29), BVD-523 (B, 3µM A375 and HT29), LY-3214996 (L, 3µM A375, 10µM HT29) or the MEK inhibitor Trametinib (T, 10nM A375, 30nM HT29). Whole cell lysates were fractionated by SDS-PAGE and immunoblotted with the indicated antibodies. Li-Cor blots are shown and are representative of those used for quantification with molecular masses in kDa indicated on the left-hand side (**A & D**). Quantitative western blot analysis of ERK1 and ERK2 are shown for

A375 (**B, C**) and HT29 (**E, F**). Within each experimental repeat relative quantification was normalised to the mean of all conditions. Results are the mean normalised blot quantification ± SEM, n=3 experiments p values p<0.05 **, p<0.01 *** p<0.001 ****using one-way analysis of variance with Tukey's multiple comparison test comparing each inhibitor to DMSO control.

Supp Figure 4. catERKi and dmERKi promote the loss of ERK2, but not ERK1, in Capan-1 pancreatic cancer cells. Capan-1 cells (**A-C**) were treated with DMSO (D), the ERK inhibitors Compound 27 (27, 1µM), SCH772984 (S, 3µM), GDC-0994 (G, 10µM), BVD-523 (B, 10µM), LY-3214996 (L, 10µM) or the MEK inhibitor Trametinib (T, 30nM). Whole cell lysates were fractionated by SDS-PAGE and immunoblotted with the indicated antibodies. Li-Cor blots are shown and are representative of those used for quantification with molecular masses in kDa indicated on the left-hand side (**A**). Quantitative western blot analysis of ERK1 (**B**) and ERK2 (**C**) are shown. Within each experimental repeat relative quantification was normalised to the mean of all conditions. Mean values +/- SEM are shown, n = 3. *, P < 0.05; **, P < 0.01; ***, P < 0.001; ****, P < 0.0001 using one-way ANOVA and Tukey post hoc test, comparing each compound with DMSO treatment.

Supp Figure 5. Both catERKi and dmERKi promote the polyubiquitylation of ERK2
HCT116 cells were treated with Cmpd 27 (100nM), BVD-523 (3µM), GDC0994 (3µM), SCH772984 (100nM), Selumetinib (100nM) or Trametinib (10nM) for 4 hours. **A** Cells were lysed in TG-lysis buffer and a portion retained as input whole cell lysate (WCL) fractionated by SDS-PAGE and immunoblotted with the indicated antibodies. **B** Equal quantities of input lysates were incubated with GST-Dsk2 or GST-Dsk2ΔUBA beads. Proteins were eluted from the beads after washing by boiling in 1xSB, fractionated by SDS-PAGE and immunoblotted with the ERK1/2 antibody 137F5. Results are from a single experiment; identical results were obtained in 3 replicate experiments.

Supp Figure 6. The NAE inhibitor MLN4924 inhibits BVD-523 induced ERK2 ubiquitylation.

HCT116 cells were treated with 3μM BVD-523 with or without 300nM MLN4924 for the indicated times. **A** Cells were lysed in TG-lysis buffer and a portion retained as input whole cell lysate (WCL) fractionated by SDS-PAGE and immunoblotted with the indicated antibodies. **B** Equal quantities of input lysates were incubated with GST-Dsk2 or GST-Dsk2ΔUBA beads. Proteins were eluted from the beads after washing by boiling in 1xSB, fractionated by SDS-PAGE and immunoblotted with the ERK1/2 antibody 137F5. Results are from a single experiment; identical results were obtained in 3 replicate experiments.

A

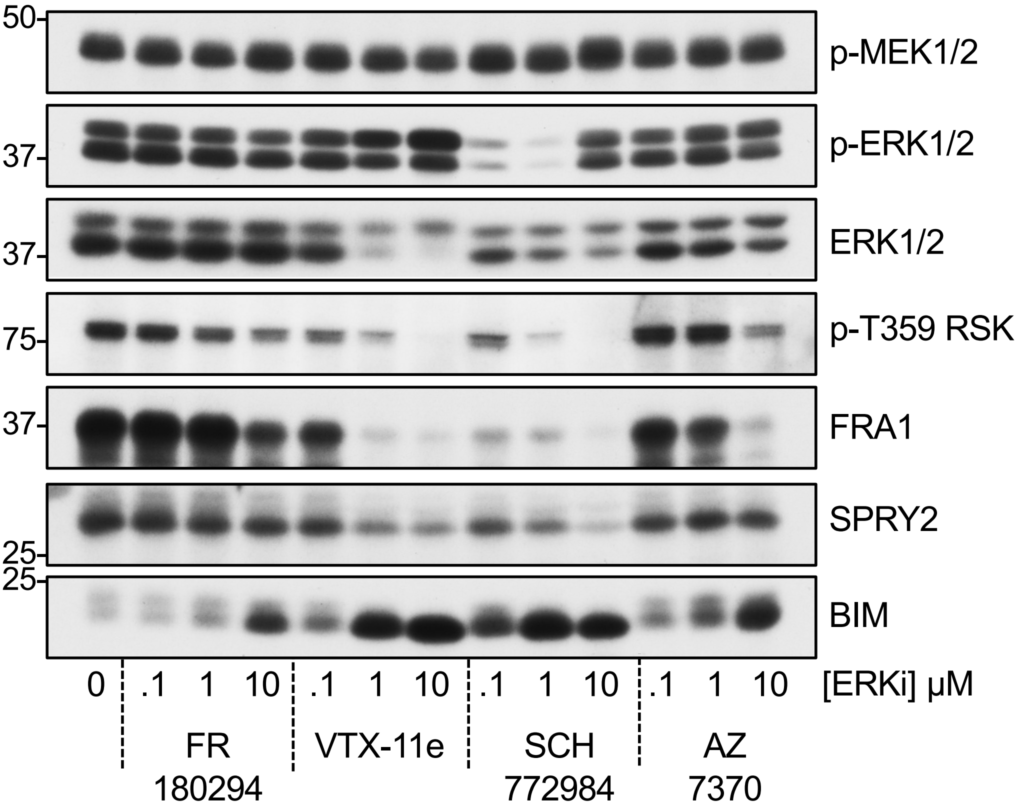
50-
37-
37-
75-
37-
25-
25-
p-MEK1/2
p-ERK1/2
ERK1/2
p-T359 RSK
FRA1
SPRY2
BIM
0 .1 1 10 .1 1 10 .1 1 10 .1 1 10 [ERKi] µM
FR 180294
VTX-11e
SCH 772984
AZ 7370

B

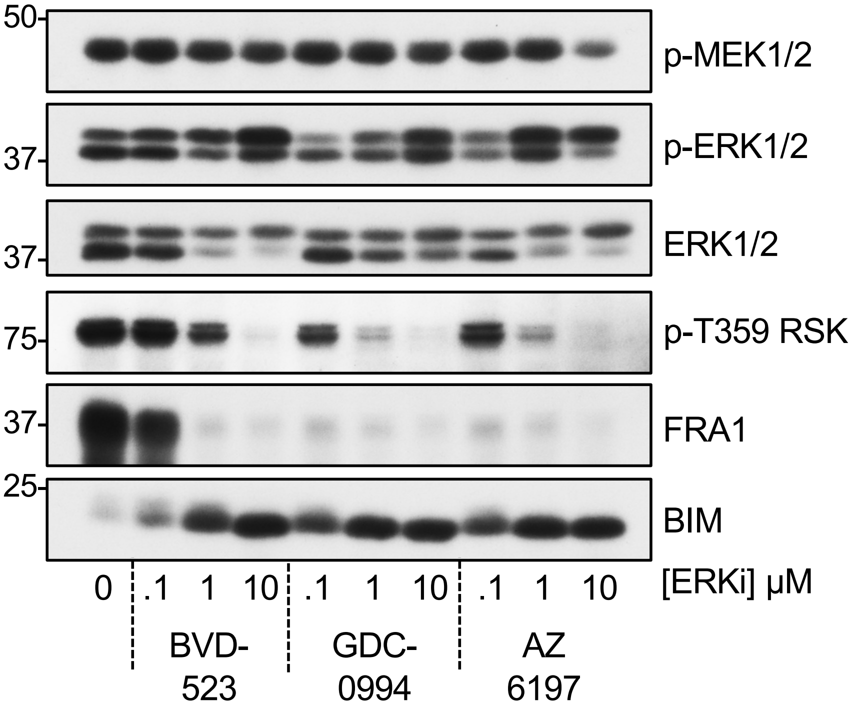
50-
37-
37-
75-
37-
25-
p-MEK1/2
p-ERK1/2
ERK1/2
p-T359 RSK
FRA1
BIM
0 .1 1 10 .1 1 10 .1 1 10 [ERKi] µM
BVD-523
GDC-0994
AZ 6197

A

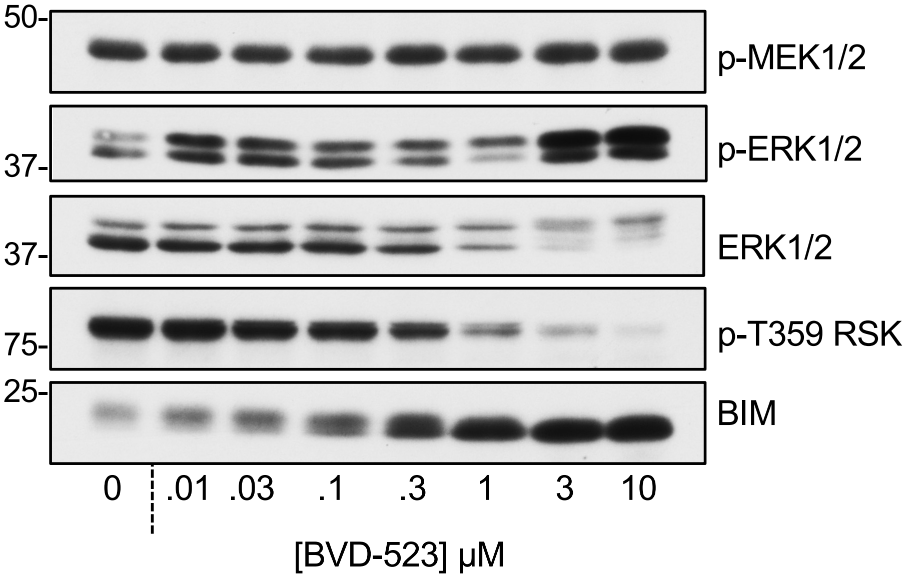
50-
37-
37-
75-
25-
p-MEK1/2
p-ERK1/2
ERK1/2
p-T359 RSK
BIM
0
.01
.03
.1
.3
1
3
10
[BVD-523] μM

B

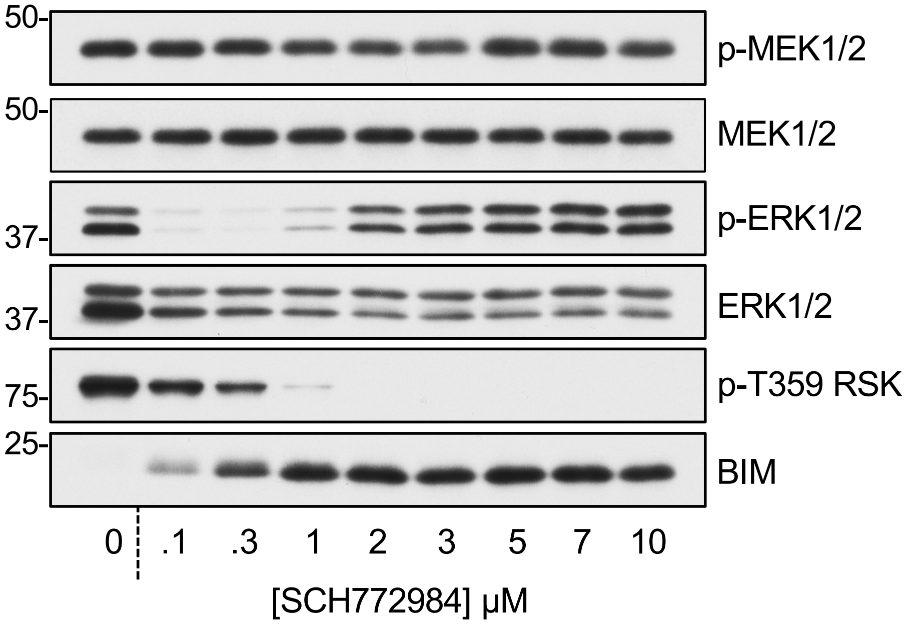
50-
50-
37-
37-
75-
25-
p-MEK1/2
MEK1/2
p-ERK1/2
ERK1/2
p-T359 RSK
BIM
0
.1
.3
1
2
3
5
7
10
[SCH772984] μM

Supp Figure 3

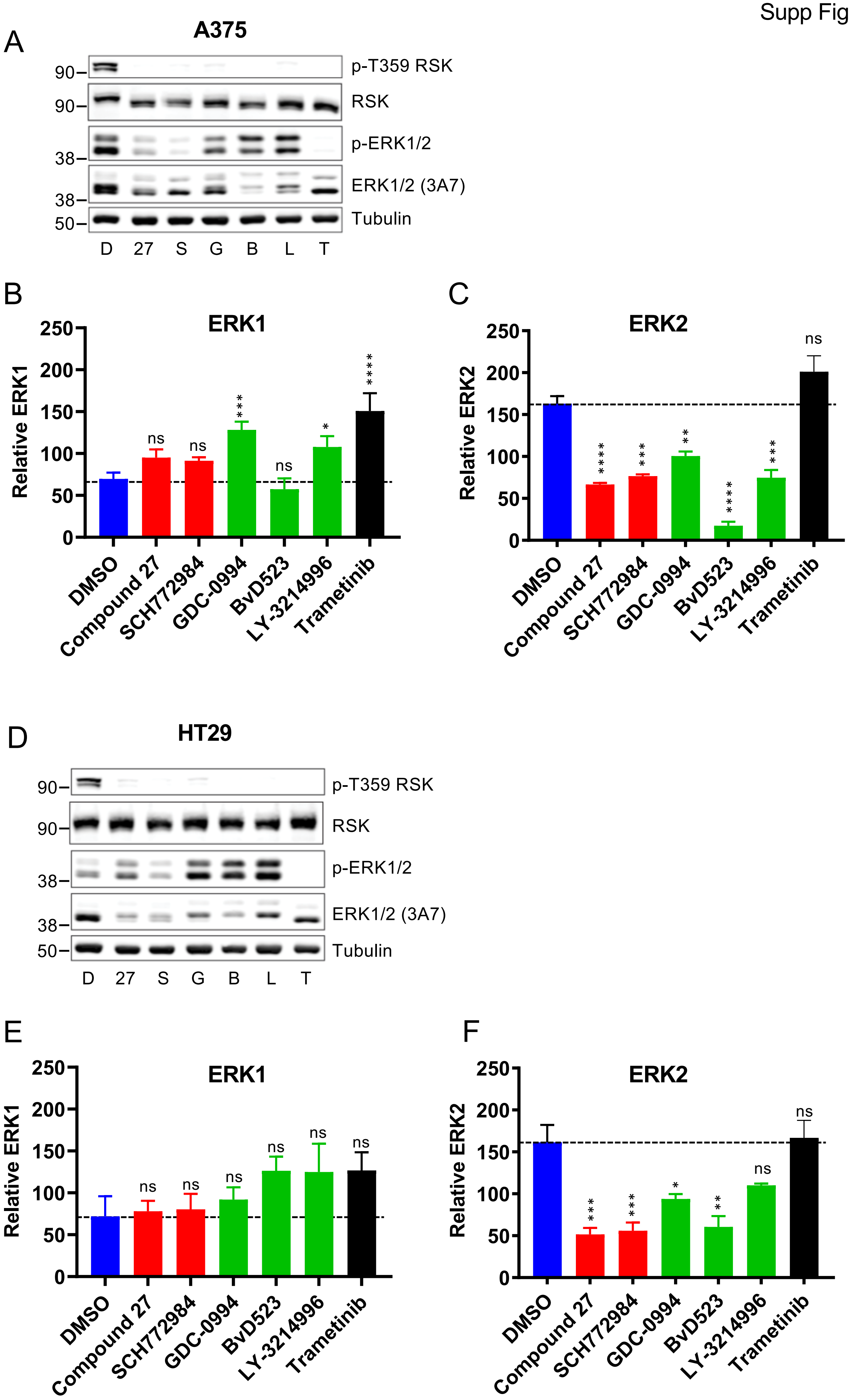

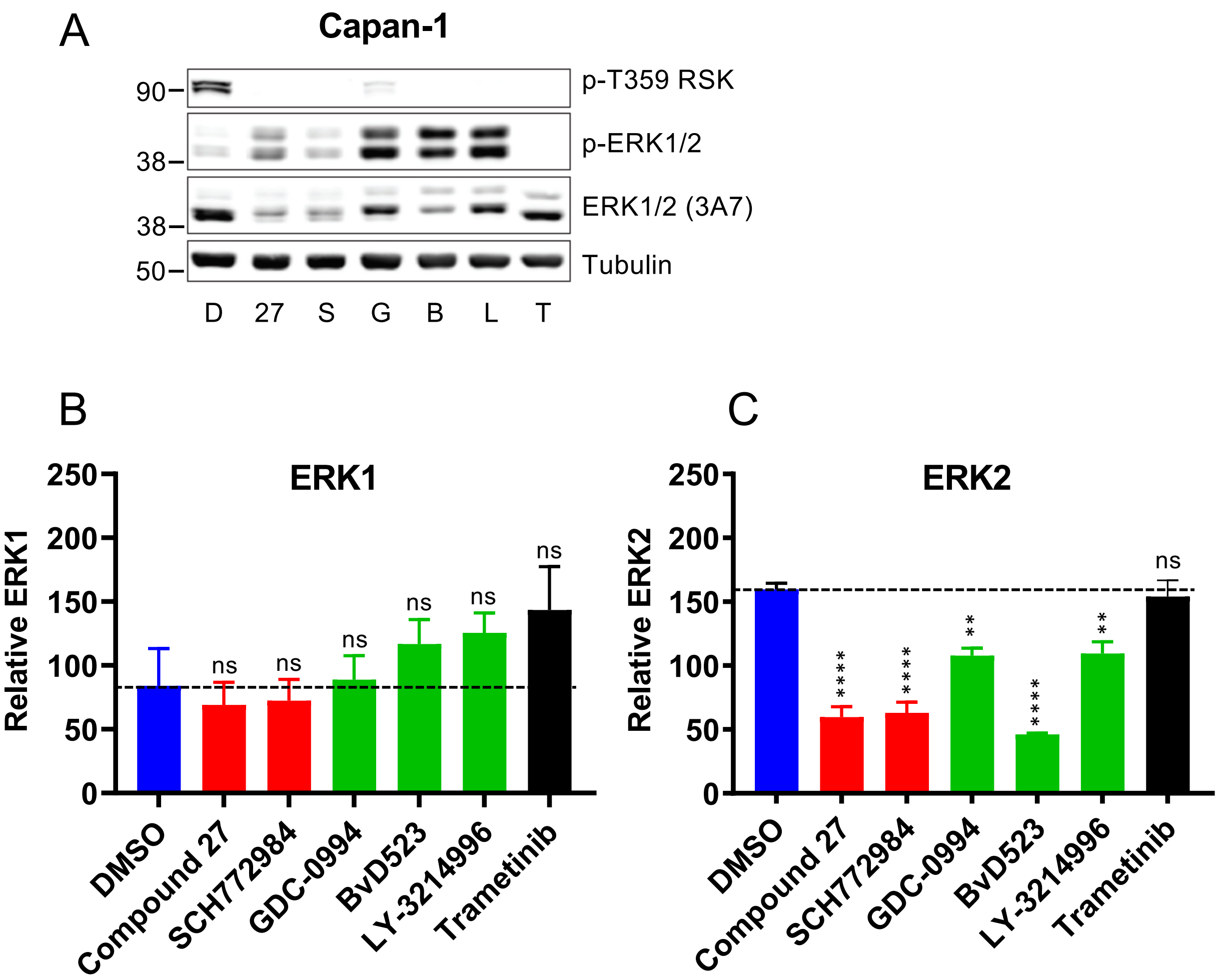
A
Capan-1
90
38
38
50
p-T359 RSK
p-ERK1/2
ERK1/2 (3A7)
Tubulin
D 27 S G B L T
B
ERK1
Relative ERK1
250
200
150
100
50
0
ns
DMSO
Compound 27
SCH772984
GDC-0994
BvD523
LY-3214996
Trametinib
C
ERK2
Relative ERK2
250
200
150
100
50
0
****
**
ns
DMSO
Compound 27
SCH772984
GDC-0994
BvD523
LY-3214996
Trametinib

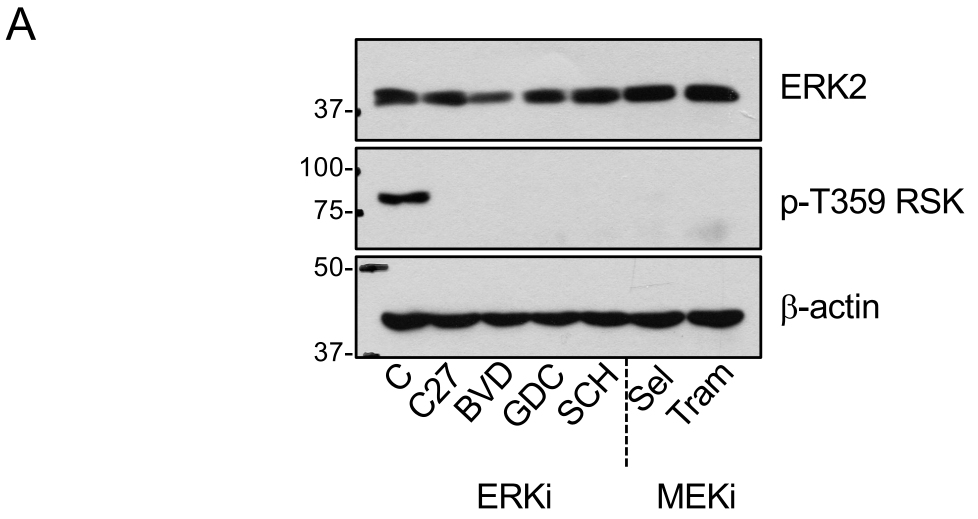
A
ERK2
p-T359 RSK
β-actin
37-
100-
75-
50-
37-
C
C27
BVD
GDC
SCH
Sel
Tram
ERKi
MEKi

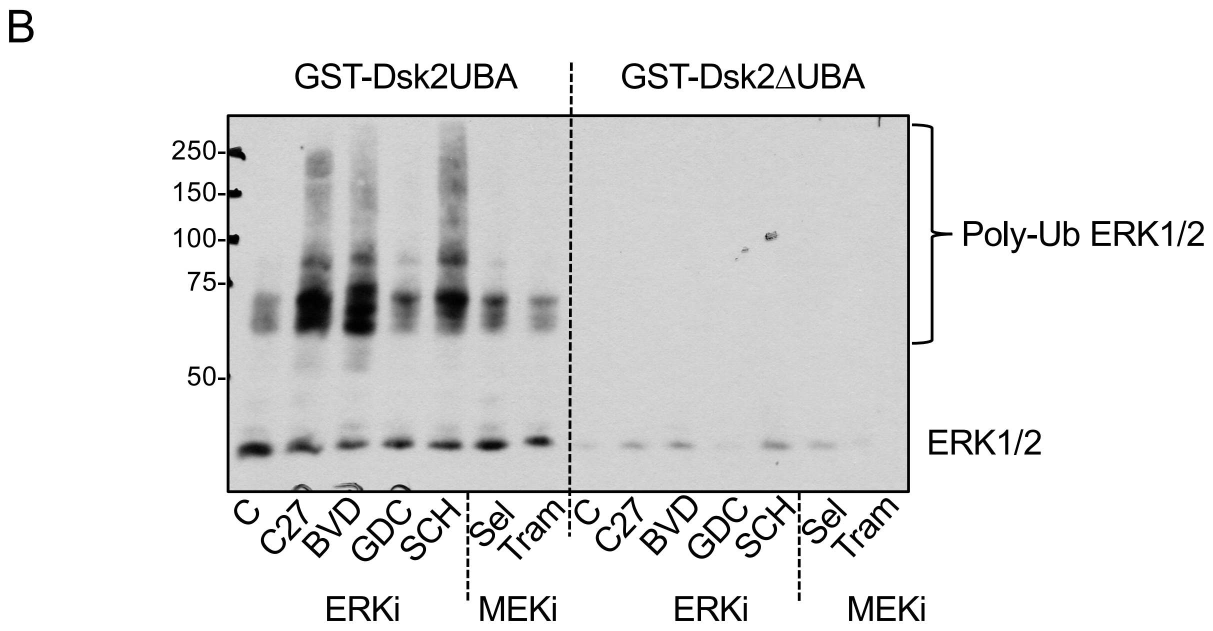
B
GST-Dsk2UBA
GST-Dsk2ΔUBA
250-
150-
100-
75-
50-
Poly-Ub ERK1/2
ERK1/2
C
C27
BVD
GDC
SCH
Sel
Tram
C
C27
BVD
GDC
SCH
Sel
Tram
ERKi
MEKi
ERKi
MEKi

A

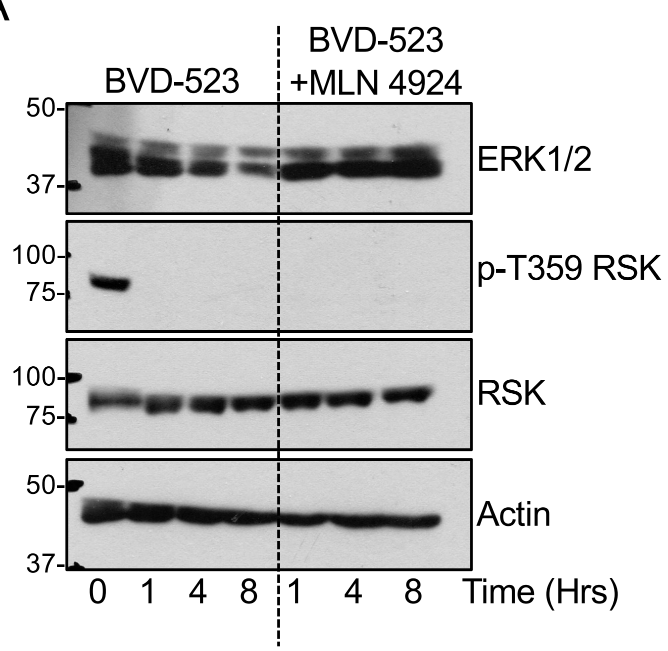
BVD-523
BVD-523
+MLN 4924
50-
37-
ERK1/2
100-
75-
p-T359 RSK
100-
75-
RSK
50-
37-
Actin
0 1 4 8 1 4 8 Time (Hrs)

B

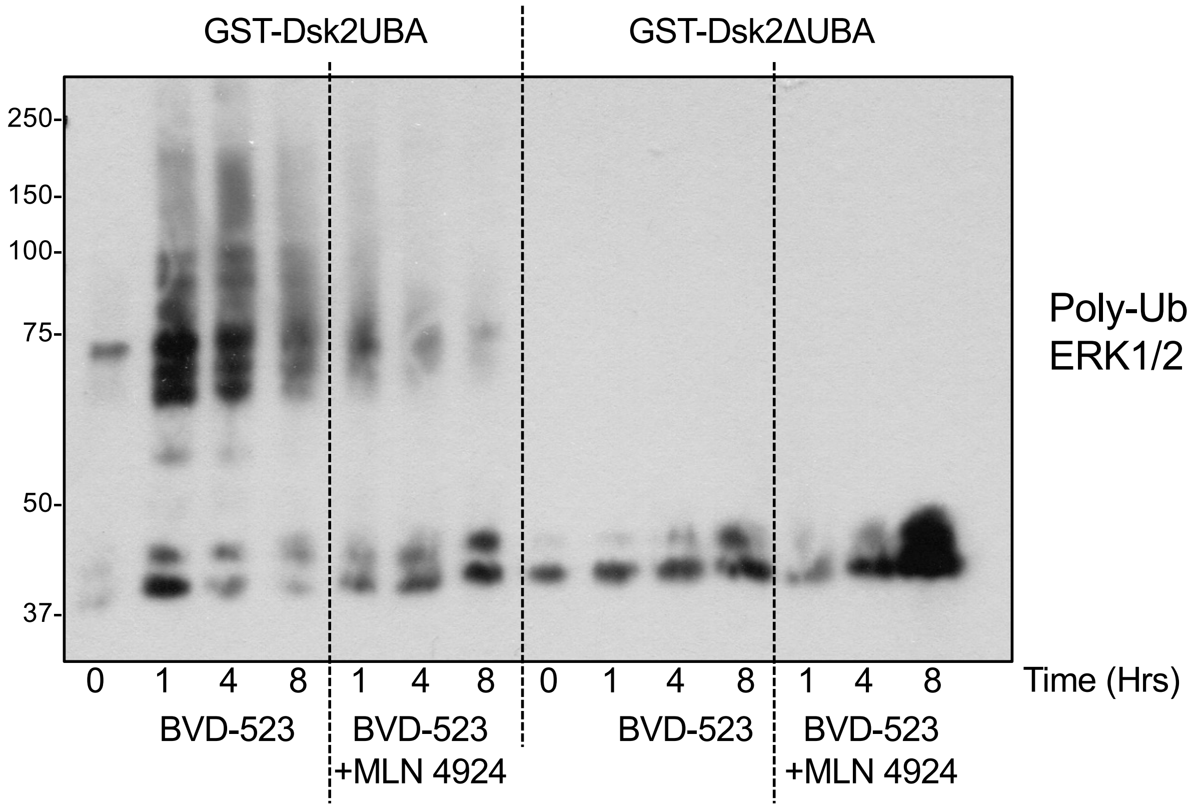
GST-Dsk2UBA
GST-Dsk2ΔUBA
250-
150-
100-
75-
50-
37-
Poly-Ub
ERK1/2
0 1 4 8 1 4 8 0 1 4 8 1 4 8 Time (Hrs)
BVD-523
BVD-523
+MLN 4924
BVD-523
BVD-523
+MLN 4924
